# Bi-nostril transseptal endoscopic approach to the sphenoidal gate. A new technique

**DOI:** 10.1007/s00701-017-3193-1

**Published:** 2017-05-12

**Authors:** Ihsan Abbas Al-Baldawi

**Affiliations:** Department of otorhinolaryngology-skull base surgery, Neuroscience hospital, Baghdad, Iraq

**Keywords:** Transsphenoidal, Septal flaps, Endoscopic, Sellar, Transseptal, Bi nostril, Transnasal, Pituitary

## Abstract

**Background:**

There are multiple approaches to reach the sellar region using microscope or endoscope, which are the old sublabial, with its post-operative morbidities, or the uni-nostril transseptal, which has a limited working field. Another technique is the direct transnasal, which is associated with tissue sacrifice, and lastly the bi-nostril (one side transseptal-other side transnasal), aiming to preserve one of the septal flaps.

**Method:**

We have 27 case studies using a new technique in which we access the sphenoid endoscopicaly through the nasal septum from both nostrils under the mucoperichondrial-periosteal flap.

**Conclusion:**

This new technique provides easy way of wide exposure to the sphenoidal gate, with easier closure of the operative field.

**Electronic supplementary material:**

The online version of this article (doi:10.1007/s00701-017-3193-1) contains supplementary material, which is available to authorized users.

## Introduction

Many surgical approaches have been advocated to reach the sphenoidal gate. Schloffer H. was the first to describe reaching the pituitary gland through the sphenoidal gate via lateral rhinotomy approach [8]. There is also the old sublabial approach as described by Halstead and Cushing [3, 5]. Hirsch advocates reaching the sphenoidal gate through the nasal septum [6], which evolved later after the innovation of the endoscope to the transseptal endoscopic approach [10], then the direct transnasal (uni-nostril or bi-nostril approaches) [7], and lastly the bi-nostril approach (one side transseptal with other side transnasal) to preserve one side of the sepal flaps [9].

The new bi-nostril transseptal approach uses both nostrils to enter through the septum with preservation of the anterior, middle septal cartilage and bone to reach the sphenoid sinus from both sides in relatively less vascular spaces with preservation of both septal flaps for later reconstruction.

**Fig. 1:**
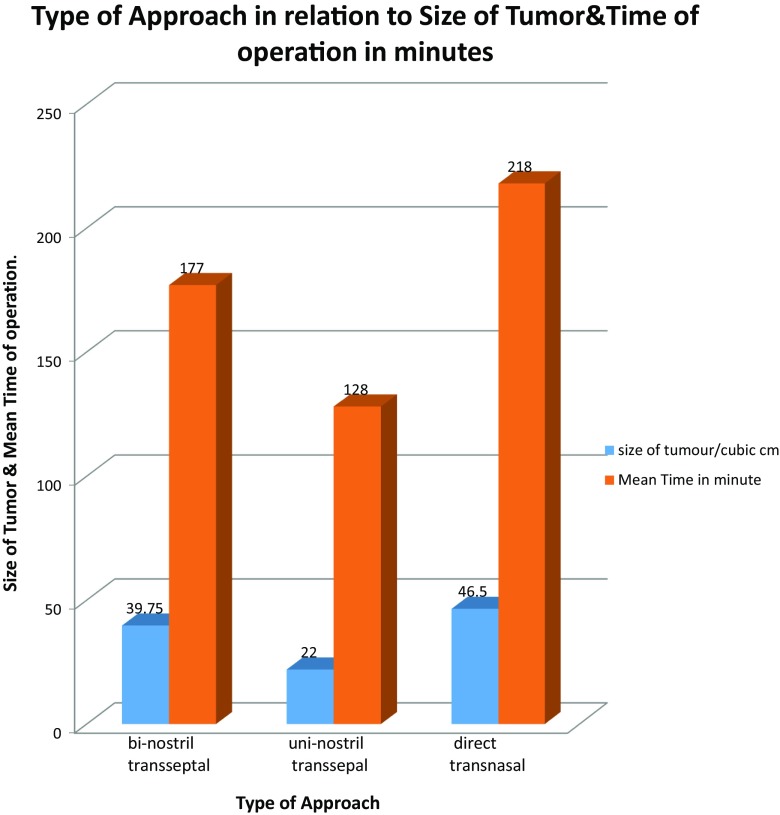
Diagram for comparing mean total anesthesia time in relation to the pre op. tumor size for the three different transsphenoidal approaches

This approach has been used in our institute (The Neuroscience Hospital in Baghdad\Iraq) since 2011. We have performed 27 cases with this approach in comparison to 42 cases performed by the uni-nostril transseptal endoscopic approach and 35 cases by the direct transnasal approach (Fig. 1).

## Relevant surgical anatomy

**Fig. 2:**
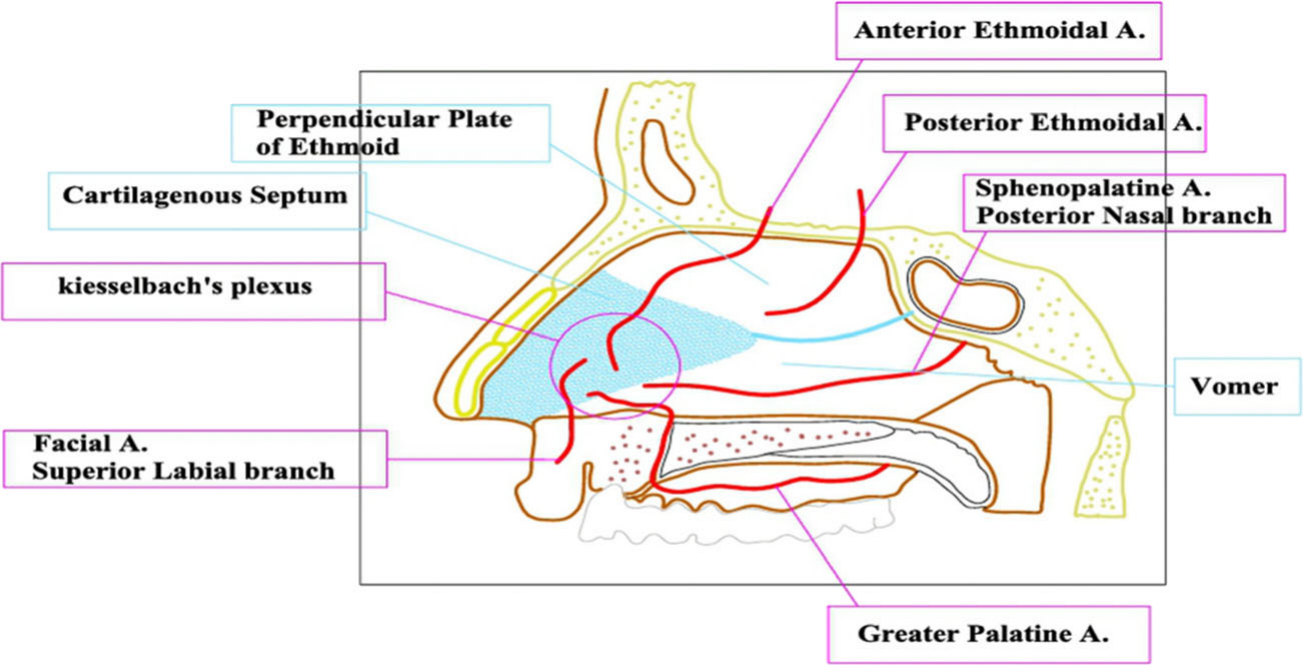
Blood supply of the nasal septum

The nasal septum composed of an anterior cartilaginous and a posterior bony structure, perpendicularly oriented wall splitting the nasal cavity into two spaces covered by thick partly mucoperichondrial and partly mucoperiosteal layer covering the cartilaginous and the boney parts respectively from both sides as one continuous layer, giving oxygenation and nutrition to the underlying cartilage and bone. They take their rich blood supply anteriorly from the four arteries which form the Kiesselbach’s vascular plexus and posteriorly from the posterior ethmoid artery and the posterior nasal branch of the sphenopalatine artery, which run on the anterior face of the sphenoid sinus running below the sinus orifice (Fig. 2). This thick septal mucosal layer can be separated from both sides away from the underlying cartilage and bone as one continuous layer.

## Description of the technique

The new technique involves applying decongestant to the nasal cavity then saline infiltration of the septal submucosal space followed by non-opposing anterior septal incisions. Then the mucosal flaps are elevated to the anterior wall of the sphenoid sinus and carrying on just lateral to the sphenoid ostium preserving their posterior pedicle with its vascular supply and stop elevation superiorly 1 cm below the level of the cribriform.

**Fig. 3:**
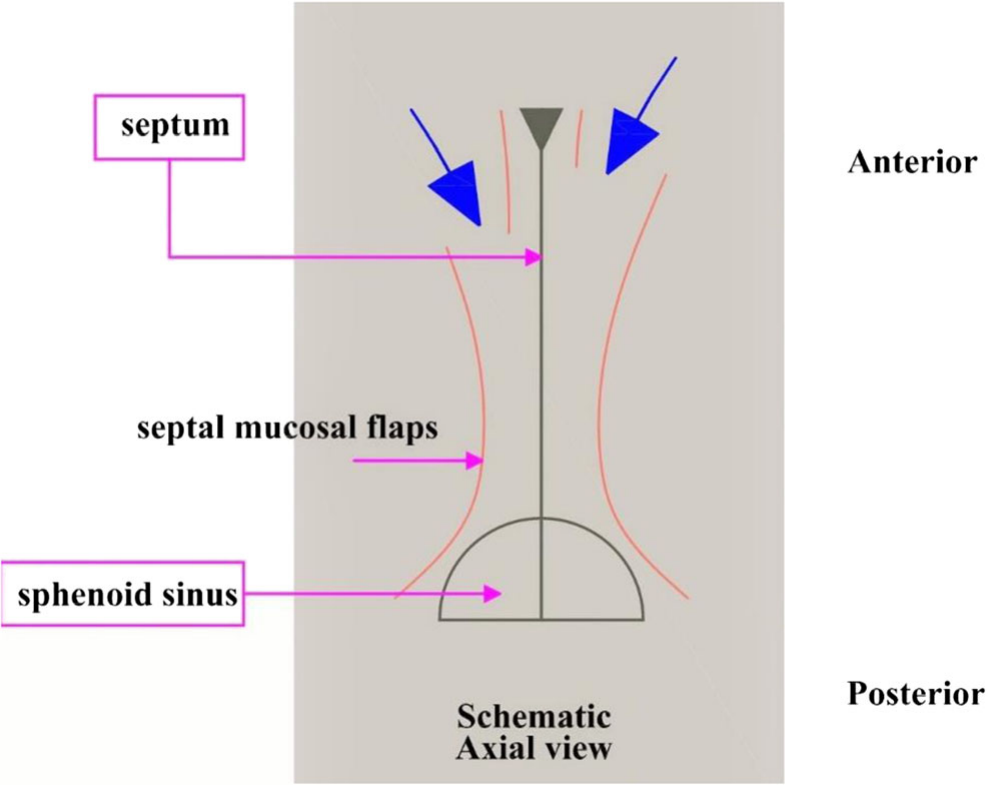
Site of entry (*blue arrows*) after a bilaterally non opposing anterior septal incisions and flaps elevation

On the floor of the nose we could extend our dissection to a limit that gives enough space for the insertion of the speculum hence creating a bilateral sub mucosal tunnel spaces (Fig. 3).

**Fig. 4:**
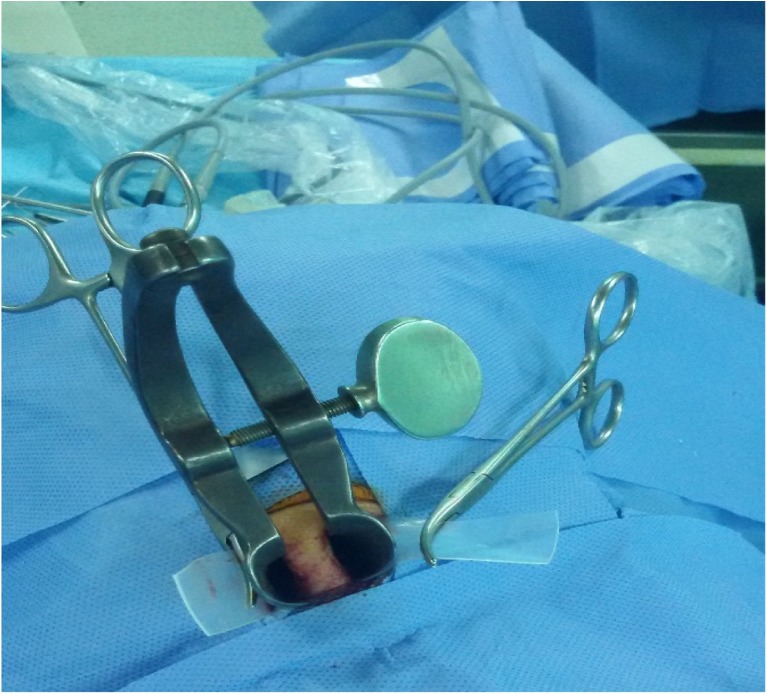
the nasal or sublabial pituitary speculum blades slides over the inner surface of the Silastic sheets

The silastic sheets are fashioned to take the shape of the tunnel to prevent overriding of the septal flaps in the field of our work and protect the flaps from any damage could be caused by the speculum blades, which slide between the silastic sheets and the septum (Fig. 4).

**Fig. 5:**
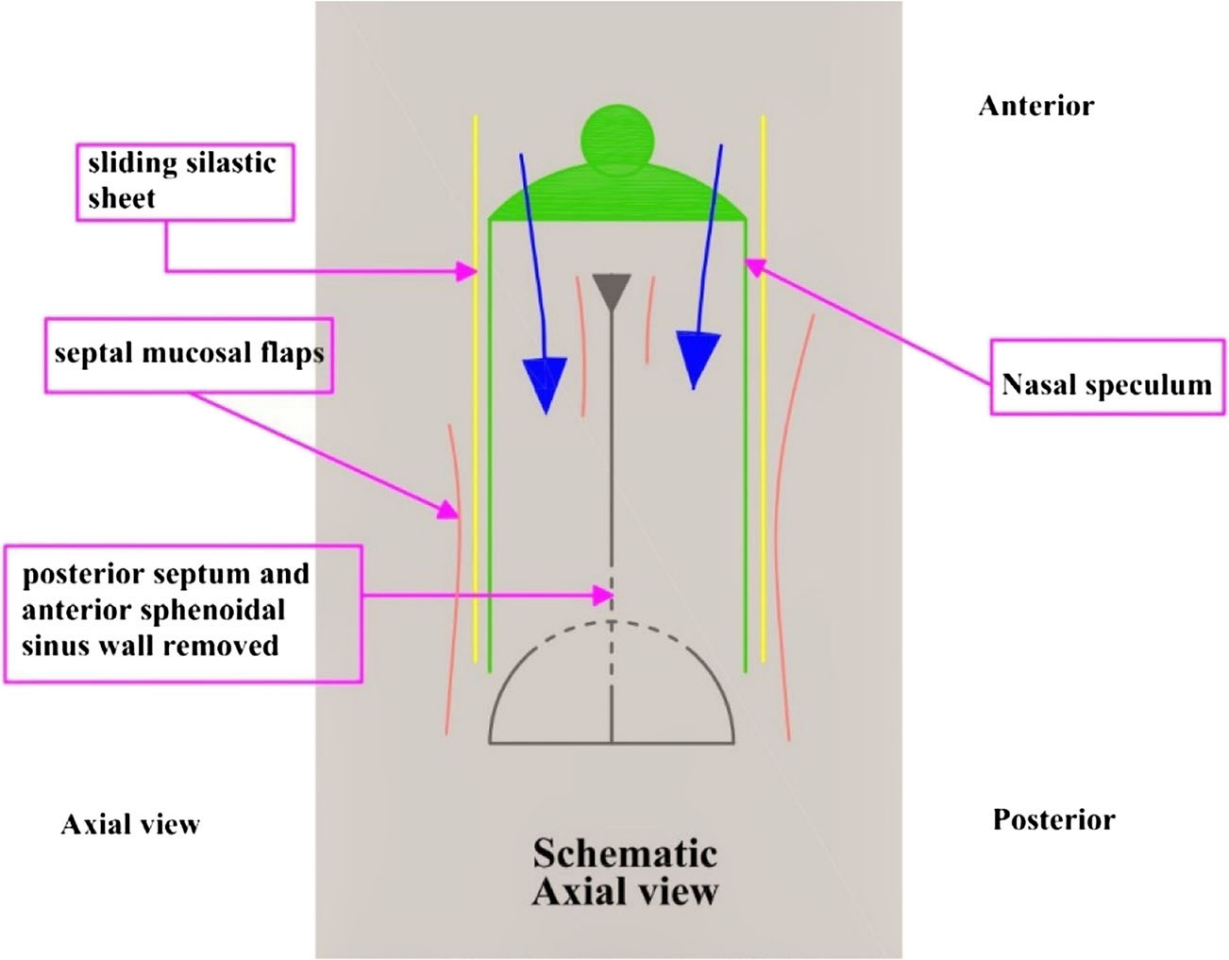
Inserting blades of nasal or sublabial pituitary speculum (*in green*) over the sliding Silastic sheets (*in yellow*) pushing septal mucosal flaps laterally (*in orange*), with removal of a small posterior bony septum and the anterior wall of sphenoid sinus

The next step would be removal of the most posterior bony nasal septum and the bony rostrum between both sphenoidal ostei (Fig. 5).

**Fig. 6:**
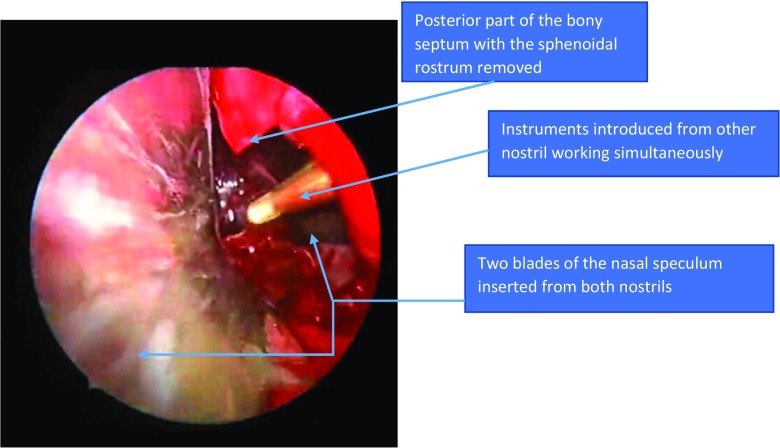
Working synonymously from both sides with different angles of work from within the nasal septum

This permits the introduction of the endoscope from one nostril and the surgical instruments through the other nostril with different viewing and working angles (Fig. 6, Videos 1 and 2).

**Fig. 7:**
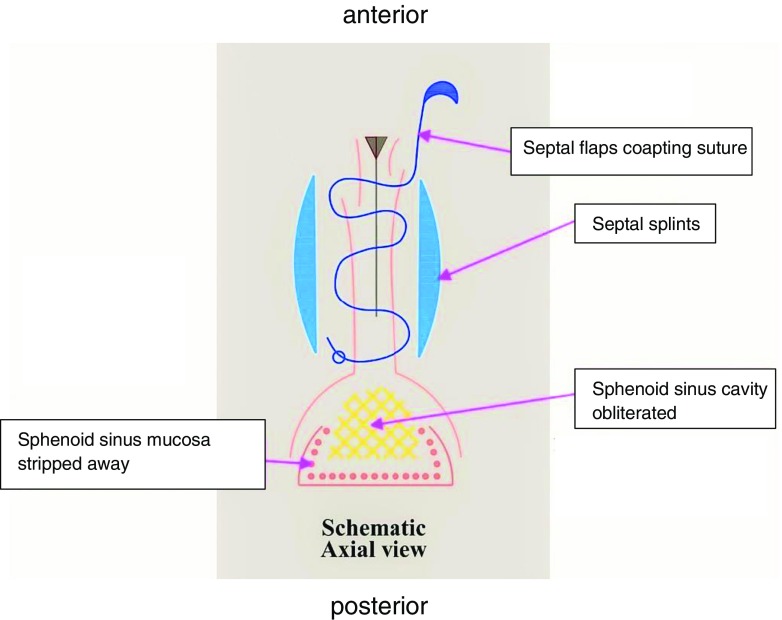
Closure obtained by coaptation absorbable sutures of both septal flaps and held by silicon septal airway splints. Sphenoid sinus mucosa stripped away with complete obliteration of its cavity (if indicated)

At the end of the operation, a proper nasal hemostasis must be achieved then a coaptation absorbable sutures and/or a bilaterally silicon septal airway splints applied to hold both septal flaps together (Fig. 7).

In certain cases of a big sphenoidal roof or posterior sphenoidal wall defects, the above method of septal mucosal flap closure repair can be used. We start by stripping off all mucosa of the sphenoid sinus then filling the sphenoid cavity by material of repair like fat, fascia, cartilage or bone before applying the above flap closure technique illustrated in Fig. 7, keeping it in a well vascularized closed space, permitting a better healing of our reconstruction post operatively.

The new technique provides wide access with different viewing and working angles from both nostrils (Fig. 5, Videos 1 and 2), less bleeding and easier to perform. It also offers enough space for work using a four hands technique [1]. This is specifically important during the control of active bleeding during surgery [2].

The bi-nostril transseptal working angles allows an extended transsphenoidal work without the need to do ethmoidectomy and middle turbinate lateralisation.

This approach also permits preservation of the anterior cartilaginous septum when working on both sides of the cartilage without the need to remove or push the cartilage to the other side. In conditions where there is no marked posterior bony septal deviation requiring extensive removal, only 2 cm from the posterior part of the bony septum with the sphenoidal bony rostrum needs to be removed.

This method permits the preservation of both septal mucosal vascularized flaps for future reconstruction [4].

Follow up of patients whom underwent this approach after 4–8 weeks revealed well healed and near normal looking nasal cavities (Video 3).

## Indications

In our practice the bi nostril transseptal approach is the preferred technique to be performed for removal of small sellar tumors, which previously used to be removed by the uni-nostril transseptal approach as it gives a better exposure.

It is also preferred for less extensive giant sellar tumors, which were previously removed by the bi-nostril direct transnasal approach, due to its shorter operative anesthesia time and the easiness of the reconstruction (Fig. 1).

## Limitations

The bi-nostril transseptal approach could not be applied in cases of extensive sellar tumor with marked lateral extension of the tumor were more lateral exposure needed due to limitations in the lateral extension of the surgical field, in addition to the inability to add the trans-pterygoid exposure to the surgical field.

## How to avoid complications

There is a possibility of a mucosal flap tear during flap elevation and silastic sheath advancement. This complication happened in 4 out of 28 cases and managed by coaptation absorbable sutures with application of a silicone septal airway splints for 2 weeks to hold both septal flaps against the underlying septum.

This complication can be avoided by meticulous flap elevation with gentle silastic sheets and nasal speculum blades advancement.

## Specific perioperative consideration

The nasal septum should be assessed for conditions like septal perforation or adhesions, which make elevation of the septal mucosal flaps more difficult.

Postoperative care includes frequent use of saline with sodium-bicarbonate nasal douche, with scheduled endoscopic local nasal debridement in the office. The silicone septal airway splints should be removed 7–14 days post operatively.

## Specific information

Patients need to be informed about the possibility of cribriform plate injury during removal of the posterior bony septum or if the surgeon misdirects the level of the sphenoid sinus through the bony septum to an upward direction, which might ends up with CSF rhinorrhea. Fortunately this has not happened in our practice.

## Summary

This technique is preferable due to the following reasons:

- Easy to perform.
- Less operative anesthesia time.
- Provides wide exposure.
- The possibility of using four hands.
- Less tissue sacrifice with less damage to the nasal mucosa
- Less bleeding at the surgical site, which in turn results in less incidence of post op hemorrhage.
- Protection, preservation and easier harvestation of the nasoseptal flaps [10], to be used for reconstruction if needed.
- No nasal packing required.
- Easier operative field closure and defects reconstruction.
- Post op near normal nasal cavity.

## Electronic supplementary material

Video 1 Intra operative video shows working from both nostrils within the nasal septum (MP4 2903 kb)

Video 2 Intra operative video shows control of active bleeding (MPG 8335 kb)

Video 3 25 days post op reveals near normal nasal cavity (MP4 14,516 kb)
